# Supraventricular Tachycardia Associated With Transdermal Scopolamine: A Case of Commonalities Leading to an Uncommon Toxicity

**DOI:** 10.1016/j.cjco.2023.12.004

**Published:** 2023-12-08

**Authors:** Nicholas Huerta, Jack Stylli, Samantha Mahar, Abhinandan Chittal, Daniel Grove

**Affiliations:** a Internal Medicine Residency Program, MedStar Union Memorial Hospital, Baltimore, Maryland, USA; b Georgetown University School of Medicine, Washington, DC, USA; c Department of Pulmonary and Critical Care Medicine, MedStar Union Memorial Hospital Baltimore, Maryland, USA; d Department of Pulmonary and Critical Care Medicine, Allegheny General Hospital, Pittsburgh, Pennsylvania, USA

**Scopolamine is a commonly used medication that is typically thought of as a benign drug. However, numerous adverse reactions to it can occur, which are often overlooked. Additionally, specific patient populations may be at higher risk of adverse events. Here, we present a case of unstable supraventricular tachycardia (SVT) associated with the use of 1 mg transdermal scopolamine in a hospitalized patient. We postulate that alterations in scopolamine metabolism from renal impairment and drug-drug interactions led to increased serum drug concentrations and precipitated the SVT.**

Scopolamine is approved by the US Federal Drug Administration to treat postoperative nausea and vomiting and motion sickness. The medicine also is used for several off-label indications, including, but not limited to, chemotherapy-induced nausea and vomiting, gastrointestinal spasms, excessive respiratory secretions, and depression. The mechanism of action of scopolamine is through competitive inhibition of the acetylcholine muscarinic receptors. These receptors are located in both the central and peripheral nervous systems. Peripherally, these receptors are present in many organ systems (cardiac, pulmonary, urinary, etc.) and activation leads to canonical parasympathetic responses. The desired anti-nausea effect of scopolamine is primarily mediated through inhibition of neural transmission from both the vestibular nuclei and the reticular formation. Given the ubiquity of muscarinic receptors and the myriad of physiological effects, many adverse reactions can occur. The transdermal route of scopolamine administration is believed to minimize the risk of anticholinergic toxicity. To our knowledge, no prior reports have been made of SVT associated with transdermal scopolamine. This case discusses the pharmacology of scopolamine and mechanisms of SVT, and provides several lines of evidence supporting the theory that the SVT was precipitated by the 1 mg of transdermal scopolamine.

## Case Presentation

A 62-year-old woman with human immunodeficiency virus infection and acquired immunodeficiency syndrome (CD4 = 4 cell/mm^3^) presented to the emergency department with complaints of a productive cough for 1 week. On arrival, she was hypoxic, chest radiography showed left-sided lingular airspace opacification, and she was found to have oropharyngeal candidiasis. Additionally, laboratory data revealed that she had acute kidney injury with a glomerular filtration rate of 26 mL/min (compared to a baseline glomerular filtration rate > 90 mL/min per 1.73 m^2^). She was admitted to a general medical unit for severe community-acquired pneumonia. Antimicrobial therapy was initiated with levofloxacin (due to penicillin allergy) for community-acquired pneumonia, trimethoprim-sulfamethoxazole (TMP-SMX) was initiated for pneumocystis jirovecii prophylaxis, and fluconazole was initiated for oropharyngeal candidiasis. For her generalized nausea, she was prescribed transdermal scopolamine 1 mg/72 h. In the 2 days after receiving scopolamine, the patient began to complain of visual disturbances and dried secretions, and she became hyperthermic to 38.3°C.

Additional medications included albuterol-ipratropium, budesonide, montelukast, guaifenesin, pregabalin, bicetegravir/emtricitabine/tenofovir, polyethylene glycol, sennoside, and subcutaneous heparin.

**Figure 1:**
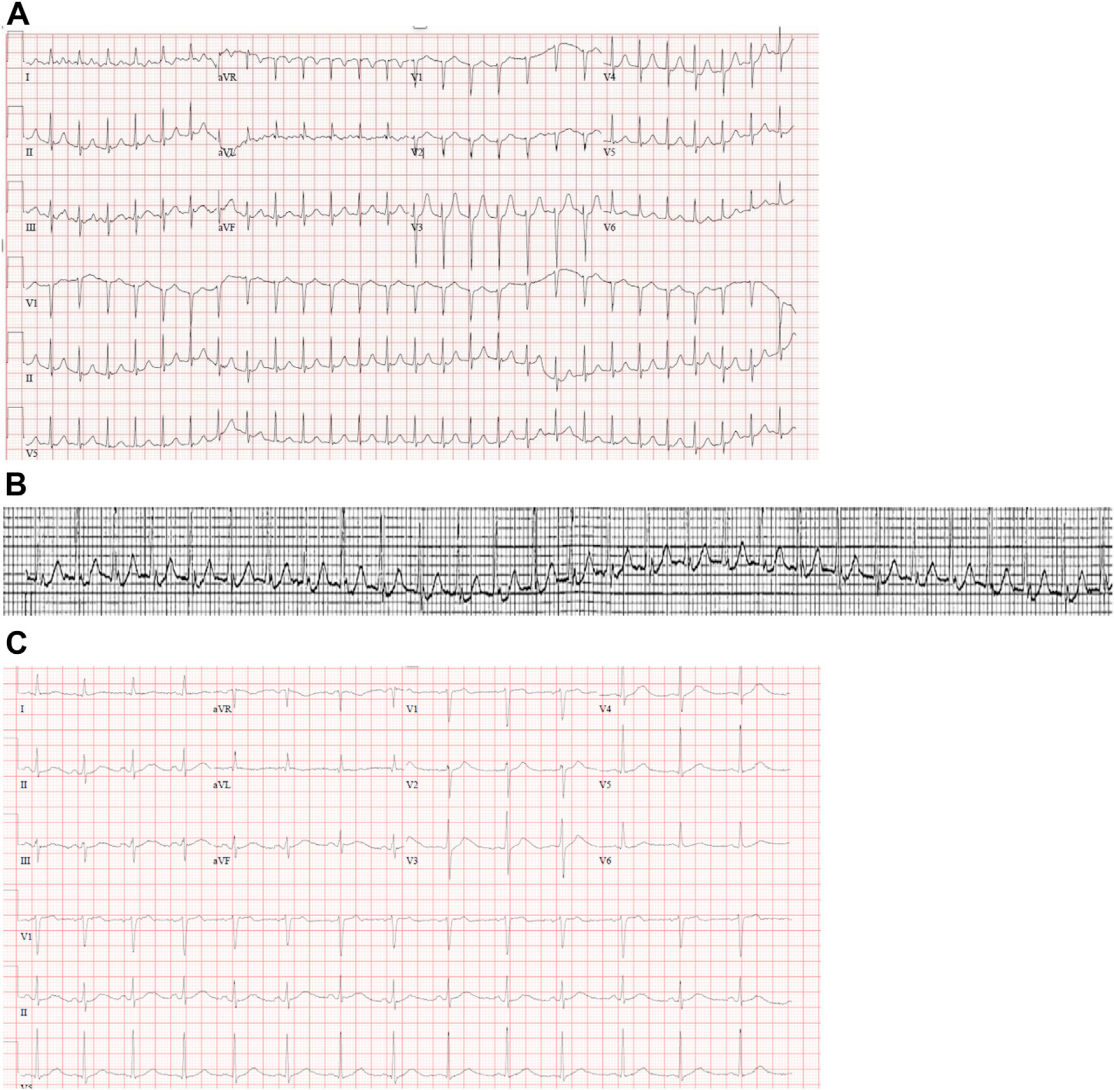
(**A**) Patient’s electrocardiogram demonstrating supraventricular tachycardia. Suspicious for typical atrioventricular nodal reentrant tachycardia because of retrograde P waves (also called pseudo-r’ waves) notable in the inferior leads. (**B**) Retrograde P waves from the continuous cardiac monitor. (**C**) Baseline electrocardiogram.

She was clinically improving on the general medical floors with decreasing supplemental oxygen requirements. Without warning or trigger, she developed SVT. Her electrocardiographic data were suspicious for atrioventricular nodal reentrant tachycardia (AVNRT) at a rate near 170 beats per minute, compared to a baseline of normal sinus rhythm (Fig. 1). She became hemodynamically unstable and was transferred to the intensive care unit. She was given 2 doses of adenosine without response and underwent emergent electrical cardioversion, with success. The scopolamine patch was removed during the event. Subsequent transthoracic echocardiogram was unremarkable. Her most recent serum potassium prior to the SVT was 4.8 mmol/L (reference: 3.5-5.0 mmol/L). The patient had no prior history of SVT, and the arrhythmia did not return after the electrical cardioversion and removal of the scopolamine patch.

## Discussion

This case is that of a patient who spontaneously developed an unstable SVT that had no clear etiology. Her electrolyte data did not reveal any abnormalities, and cardiac workup was unrevealing. The symptoms of dried secretions, visual disturbances, and hyperthermia after receiving scopolamine suggest an anticholinergic mechanism. We therefore postulate that the SVT was precipitated by transdermal scopolamine. This patient had several alterations in metabolism of scopolamine, which are discussed.

Scopolamine’s adverse effects have been described previously in varying populations, but this case brings up several important points regarding the pharmacokinetics and pharmacodynamics of scopolamine. First, scopolamine metabolism is hepatically driven via the cytochrome p-450 (CYP) system.^1^ The parent drug undergoes demethylation via the CYP3A subfamily. This subfamily is moderately inhibited by fluconazole, which was a new medication introduced to the patient during the hospitalization. *In vitro* evidence has shown that azole antifungals decrease metabolism of other drugs metabolized by the CYP3A system by 87%.^2^ Furthermore, *in vivo* human evidence on scopolamine shows that CYP3A inhibition leads to increased bioavailability.^1^ Additionally, age, sex, and genetic polymorphisms in CYP enzymes also may lead to clinically relevant alterations in anticholinergic drug metabolism.^3^

Second, scopolamine is excreted through the urine. Despite its having been used for more than 100 years, investigation into human metabolism of scopolamine has been minimal.^1^ In this patient with significant renal impairment, who was also taking a potentially nephrotoxic medication (TMP-SMX), we hypothesize that active metabolites may have accumulated. Thus, CYP inhibition from fluconazole, renal impairment, and use of TMP-SMX may have led to increased serum drug concentrations.

**Figure 2:**
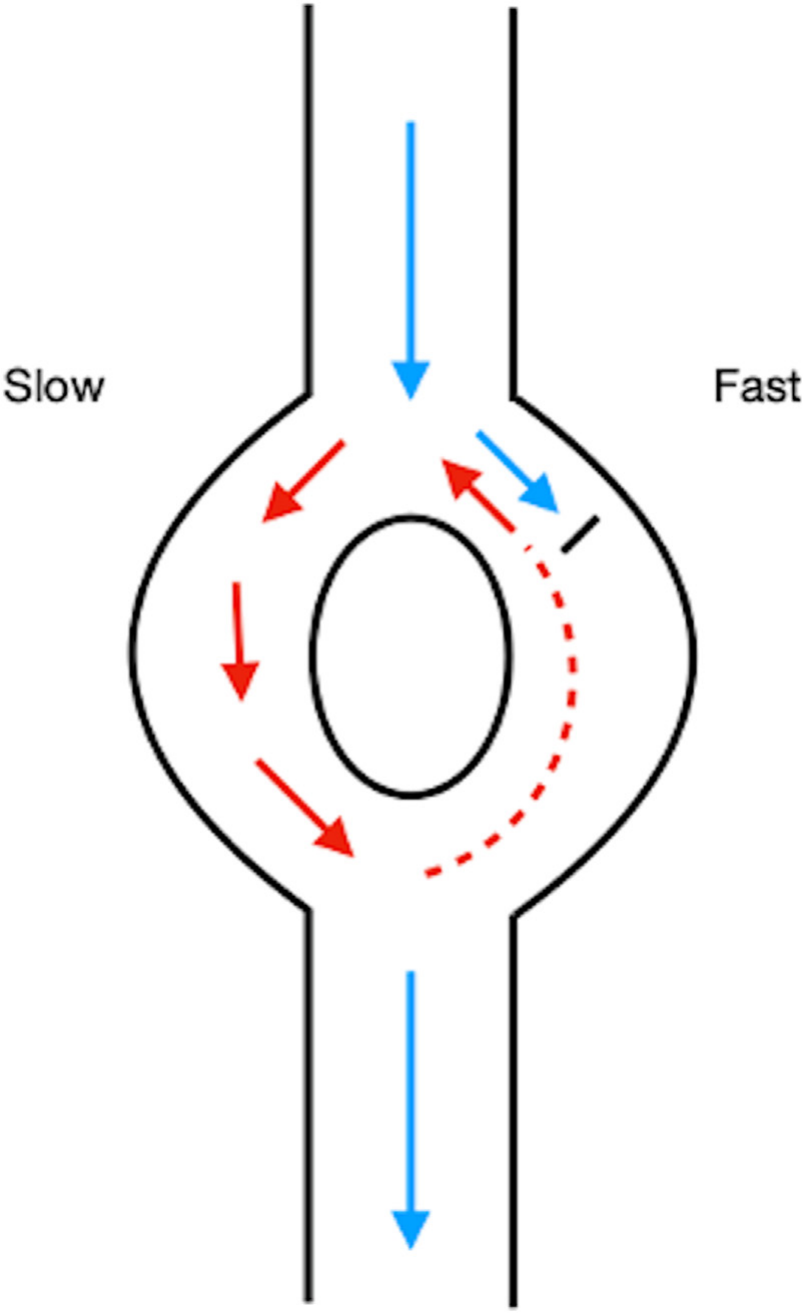
Typical atrioventricular (AV) nodal reentrant pathway. The normal electrical impulse flows anterograde to the AV node where it encounters 2 pathways: slow and fast. If the fast pathway is refractory, this blocks the impulse from going any further. This blocking can allow the impulse in the slow pathway to flow down the AV node, where it eventually conducts to the ventricle. However, the impulse can also flow retrograde through the fast pathway if it is no longer refractory. This retrograde impulse then reaches the antegrade slow pathway and establishes a circuit. This circuit, with the impulse traveling anterograde through the slow pathway and retrograde through the fast pathway, is the mechanism of typical AV nodal reentrant tachycardia.

Third, anticholinergics are known to effect cardiac chronotropy and dromotropy.^4^^,^^5^ The model anticholinergic drug atropine may induce AVNRT and is sometimes used in electrophysiologic studies to unmask dual atrioventricular node physiology. Additionally, intravenous atropine has the potential to induce AVNRT in patients previously naïve to any SVT.^5^ However, several studies have identified differing effects of atropine on atrioventricular nodal conduction.^4^, ^5^, ^6^, ^7^ These differing effects may be caused by individual variations in cardiac conduction characteristics, vagal tone, and differences in the methods used to induce AVNRT (ie, atrial extra stimuli, rapid incremental pacing, etc.). The most common mechanism of AVNRT is “slow-fast”, meaning that both anterograde conduction, through the slow pathway, and retrograde conduction, through the fast pathway, occur (Fig. 2). The mechanism by which atropine may facilitate AVNRT is a decrease in the refractory period of both the slow and fast pathways. Effects on the pathways are likely to be unbalanced, leading to a conduction differential and ultimately, increased probability of establishing a continuous circuit.^7^

Scopolamine and atropine are virtually identical molecules; the only difference is an epoxide group that is found in scopolamine. Atropine metabolism in humans leads to the production of noratropine and atropine-n-oxide, both of which have anticholinergic properties. Given the structural and functional similarities between atropine and scopolamine, a reasonable theory is that scopolamine also may lead to SVT in certain patients through similar mechanisms.

## Conclusion

The patient had no further arrhythmias, maintained hemodynamic stability, and was ultimately discharged from the hospital. This case illustrates that transdermal scopolamine, typically a clinically innocuous drug, may have nuanced anticholinergic toxicity risks in certain patient populations, particularly patients with altered CYP metabolism or decreased renal function who may not follow the standard pharmacokinetic profile. An extremely common occurrence is for hospitalized patients to experience renal impairment and receive new medications that are hepatically metabolized. Clinicians should remain vigilant when treating hospitalized patients with several new medications and be aware of the potential interactions and toxicities.

**Novel Teaching Points**

• Transdermal scopolamine may induce supraventricular arrhythmias.
• Clinicians must be aware of drug-drug interactions in hospitalized patients started on several new medications.
• Understanding drug metabolism is critical, to assess risk of toxicity.
